# Neuroglobin-Deficiency Exacerbates *Hif1A* and c-FOS Response, but Does Not Affect Neuronal Survival during Severe Hypoxia *In Vivo*

**DOI:** 10.1371/journal.pone.0028160

**Published:** 2011-12-02

**Authors:** Christian Ansgar Hundahl, Hendrik Luuk, Sten Ilmjärv, Birgitte Falktoft, Zindy Raida, Jonas Vikesaa, Lennart Friis-Hansen, Anders Hay-Schmidt

**Affiliations:** 1 Department of Clinical Biochemistry, University of Copenhagen, Bispebjerg Hospital, Copenhagen, Denmark; 2 Department of Physiology, University of Tartu, Tartu, Estonia; 3 Quretec Ltd, Tartu, Estonia; 4 Department of Neuroscience and Pharmacology, Faculty of Health Sciences, University of Copenhagen, Copenhagen, Denmark; 5 Department of Genomic Medicine, University of Copenhagen, Rigshospitalet, Copenhagen, Denmark; Hôpital Robert Debré, France

## Abstract

**Background:**

Neuroglobin (Ngb), a neuron-specific globin that binds oxygen *in vitro*, has been proposed to play a key role in neuronal survival following hypoxic and ischemic insults in the brain. Here we address whether Ngb is required for neuronal survival following acute and prolonged hypoxia in mice genetically Ngb-deficient (Ngb-null). Further, to evaluate whether the lack of Ngb has an effect on hypoxia-dependent gene regulation, we performed a transcriptome-wide analysis of differential gene expression using Affymetrix Mouse Gene 1.0 ST arrays. Differential expression was estimated by a novel data analysis approach, which applies non-parametric statistical inference directly to probe level measurements.

**Principal Findings:**

Ngb-null mice were born in expected ratios and were normal in overt appearance, home-cage behavior, reproduction and longevity. Ngb deficiency had no effect on the number of neurons, which stained positive for surrogate markers of endogenous Ngb-expressing neurons in the wild-type (wt) and Ngb-null mice after 48 hours hypoxia. However, an exacerbated hypoxia-dependent increase in the expression of c-FOS protein, an immediate early transcription factor reflecting neuronal activation, and increased expression of Hif1A mRNA were observed in Ngb-null mice. Large-scale gene expression analysis identified differential expression of the glycolytic pathway genes after acute hypoxia in Ngb-null mice, but not in the wts. Extensive hypoxia-dependent regulation of chromatin remodeling, mRNA processing and energy metabolism pathways was apparent in both genotypes.

**Significance:**

According to these results, it appears unlikely that the loss of Ngb affects neuronal viability during hypoxia *in vivo*. Instead, Ngb-deficiency appears to enhance the hypoxia-dependent response of Hif1A and c-FOS protein while also altering the transcriptional regulation of the glycolytic pathway. Bioinformatic analysis of differential gene expression yielded novel predictions suggesting that chromatin remodeling and mRNA metabolism are among the key regulatory mechanisms when adapting to prolonged hypoxia.

## Introduction

Hypoxia and ischemic injury are both characterized by reduced oxygen availability in the tissue, which is particularly detrimental for the brain (and heart) due to its very high metabolic demand [1]. Unlike the muscles, where myoglobin can store and facilitate oxygen diffusion from the blood, the brain was believed to lack a similar storage system until Burmester and coworkers [2] discovered a neuron-specific globin, which they named Neuroglobin (Ngb). Subsequently, most studies were directed at investigating Ngb's possible involvement in oxygen storage and/or intracellular diffusion of oxygen (for review [3]). However, unlike myoglobin, Ngb is expressed at much lower concentrations and the iron atom in Ngb's heme group is quickly oxidized to Fe^3+^ *in vitro*, which renders it incapable to bind oxygen again once oxygen has been released [4], [5]. In addition, the expression pattern of Ngb in the brain is restricted to a few areas, which makes Ngb an unlikely candidate for a ubiquitous oxygen storage system in the brain [6], [7], [8], [9]. Notwithstanding the marked differences from myoglobin and at least partly due to reported up-regulation of Ngb expression during hypoxia, it has been proposed that Ngb may have neuroprotective properties during hypoxia *in vitro* [10], [11] and in animal models of ischemia [10], [12], [13], [14], [15]. While the mechanism by which Ngb may protect neurons is still unknown, it has been suggested that Ngb could be an important pharmacological target in combating stroke injury [12], [14], [16], [17]. The studies conducted so far have used animal models where the expression of Ngb was manipulated by viral up/down regulation or transgenic over-expression and we are unaware of studies conducted in genetically Ngb-deficient mice.

The present study evaluates the effect of hypoxia on neuronal survival and gene regulation in Ngb-null mice leading us to the following conclusions: 1) Ngb is not vital for neuronal survival during hypoxia, but 2) Ngb deficiency has a global aggravating effect on the expression of key hypoxia regulatory genes and 3) Ngb deficiency alters the hypoxia-dependent transcriptional response of genes related to the glycolysis pathway. Furthermore, by applying a novel probe-level data analysis method to the Affymetrix Mouse Gene 1.0 ST arrays, we have estimated the differential expression significance of all known and predicted Ensembl transcripts in the mouse transcriptome thereby offering a detailed and full-scale characterization of transcriptional regulation, during hypoxia in the brain.

## Results

### Ngb-deficient mice

**Figure 1:**
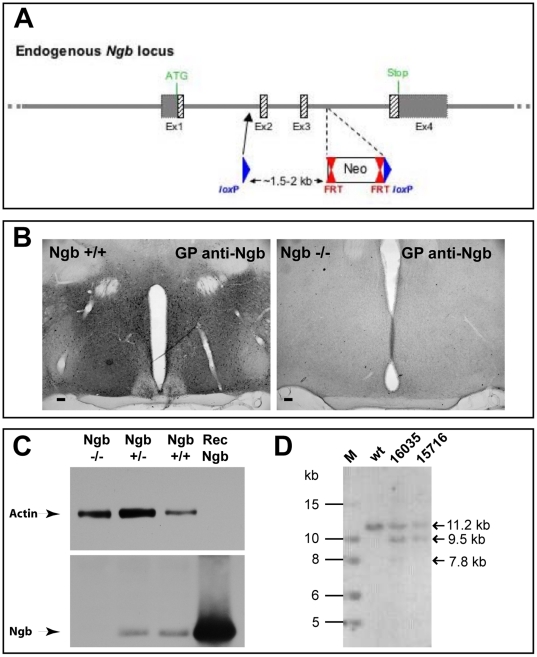
Creation of the Ngb-deficient mouse strain. **A**) the location of LoxP sites and Neo-cassette introduced in the Ngb gene by homologous recombination. **B**) immunohistochemical staining of hypothalamic tissue sections from wt and Ngb-null mice using a guinea pig anti-Ngb antibody. Strong Ngb immunoreactivity (IR) is observed in wt mice whereas IR was abolished in Ngb-null mice. **C**) Western blotting of Ngb-null (−/−), heterozygote (+/−) and wt (+/+) brains confirmed the absence of Ngb protein in Ngb-null mice. Recombinant Ngb (Rec Ngb) was used as a positive control. Actin was used as a loading control. **D**) Southern blot analysis for the validation of the heterozygous Neo-excised floxed mice. A 3′ external probe was used to confirm correct recombination and excision of Neo from the floxed allele. Predicted size of specific hybridization targets after digesting genomic DNA with BamHI-SpeI: wild-type allele 11153 bp, recombined allele 7765 bp, floxed (Flp-mediated Neo-excised) 9457 bp. Mice 16035 and 15716 are both positive for the Flp mediated Neo-deleted conditional knock-out Ngb allele. Note that mouse No. 16035 is a mosaic of Neo-excised and non-excised cells as indicated by the very weak signal from the nonexcised recombined allele. Mouse No. 15716 was used for the generation of the constitutive Ngb knockout strain. Abbreviations: 1 kb DNA-Ladder (NEB) (M); wild-type (wt); heterozygous Neo-excised floxed mice (16035 and 15716).

Ngb was targeted by introducing *LoxP* sites into the introns flanking exons 2 and 3, which were subsequently removed by mating Ngb-floxed mice with C57BL/6J Cre deleter mice (genOway) (Figure 1A, please see section “Ngb deficient mice” in Materials and Methods, for details). Deletion of Ngb mRNA and protein was confirmed by RT-QPCR (Figure S1), IHC (Figure 1B) and Western blotting (Figure 1C). Heterozygous, wild-type and homozygous Ngb-deficient mice were born in expected numbers (observed/expected: 111/93.50, 40/46.75, 36/46.75; p = 0.1997, Fisher's Exact Test) and they were normal in overt appearance, body weight, home-cage behavior and longevity. Ngb-null mice were fertile and had an average litter size of 7.5 pups at day P7.

### Effect of Ngb deficiency during hypoxia

Survival rates and behavioral responses to hypoxia were virtually identical in the wt and Ngb-null mice, and all animals (except one wt mouse) survived up to 96 hours of 7% O_2_/ 93% N_2_ hypoxia. Behavioral response to hypoxia was observed as: 1) the mice became calm and immobile within 15–30 minutes after the onset of hypoxia 2) they responded to the tapping of the cage or gentle shaking by moving around slowly and 3) were observed to feed and consume water during the experimental procedure.

### Effect of Ngb deficiency on the number of Orexin-A and Cygb-IR neurons after hypoxia

**Figure 2:**
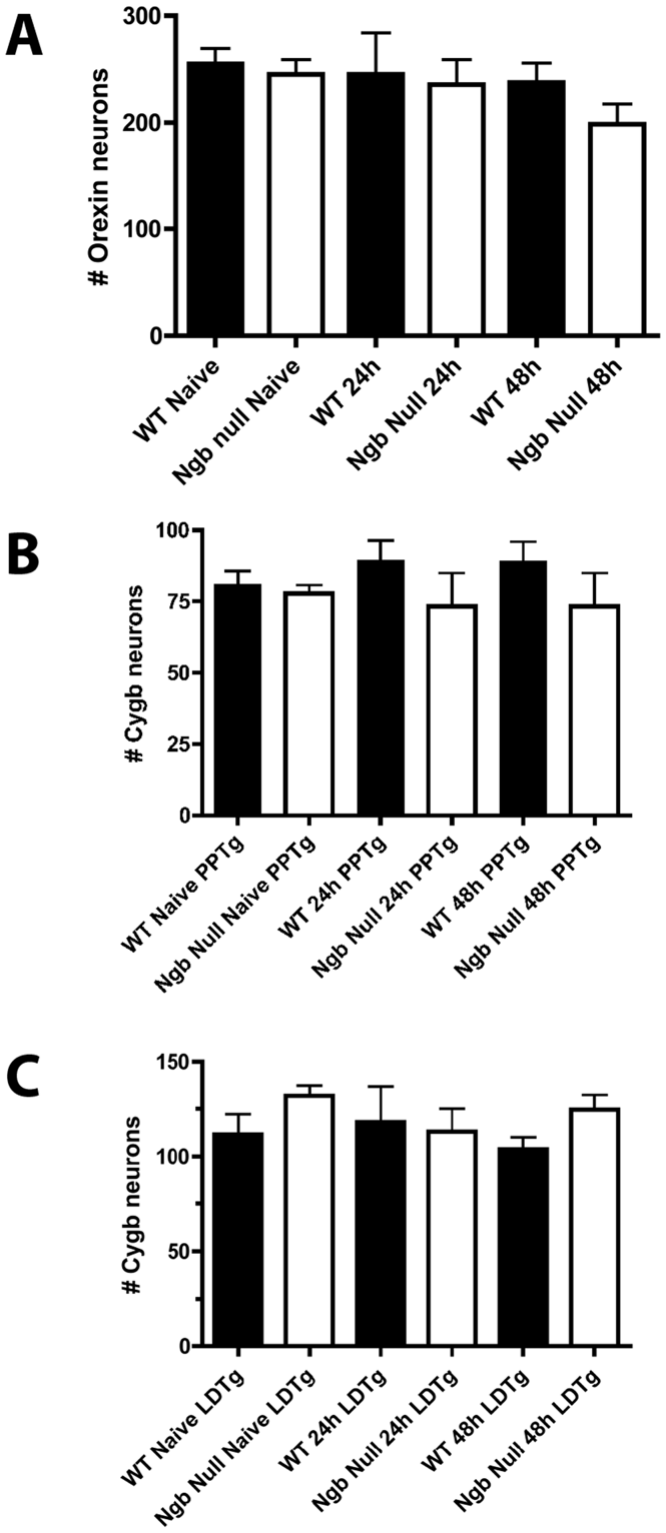
The effect of hypoxia on the viability of neurons expressing surrogate markers of Ngb in wt and Ngb-deficient mice. **A**) the number of Orexin-IR neurons in the lateral hypothalamus of naive wt and Ngb-null mice during normoxia and after 24 and 48 hours of hypoxia. No significant difference in the number of Orexin neurons was observed between the two genotypes (n = 3–6, p>0.05). **B–C**) the number of Cygb-IR neurons in PPTg (**B**) and LDTg (**C**) of wt (black bars) and Ngb-null (white bars) in naive mice and mice exposed to 24 h and 48 h of hypoxia. No significant difference in the number of Cygb-IR neurons was observed between the two genotypes (n = 3–6, p>0.05). Abbreviations: pedunculopontine tegmental nucleus (PPTg); laterodorsal tegmental nucleus (LDTg).

We have previously shown a high degree of co-expression of Ngb, Orexin-A and Cygb [8], [19]. It was estimated that 85+/−1.9% of the Orexin-A neurons, which were counted in the lateral hypothalamus, co-stored Ngb and between 46+/−4.9% and 58+/−3.6% of the Cygb neurons in the hindbrain pontine nuclei co-stored Ngb. It is therefore valid to use Orexin-A and Cygb-IR as surrogate markers for endogenously Ngb-positive neurons, which enabled us to estimate the effect of Ngb-deficiency on neuronal survival in the Ngb-null mice. Assuming Ngb to be necessary for the survival of the neurons a reduction in the number of Orexin-A and Cygb neurons should be observed in the aforementioned brain regions in Ngb-null mice following hypoxia. We found no significant differences in the number of Orexin-A-IR neurons and Cygb-IR neurons when comparing wt and Ngb-null mice during normoxia or after exposure to 24 hours or 48 hours of hypoxia (all p>0.05, Mann Whitney test) (Figure 2A–C).

### Effect of Ngb deficiency on c-FOS and activated caspase-3 expression following acute hypoxia

**Figure 3:**
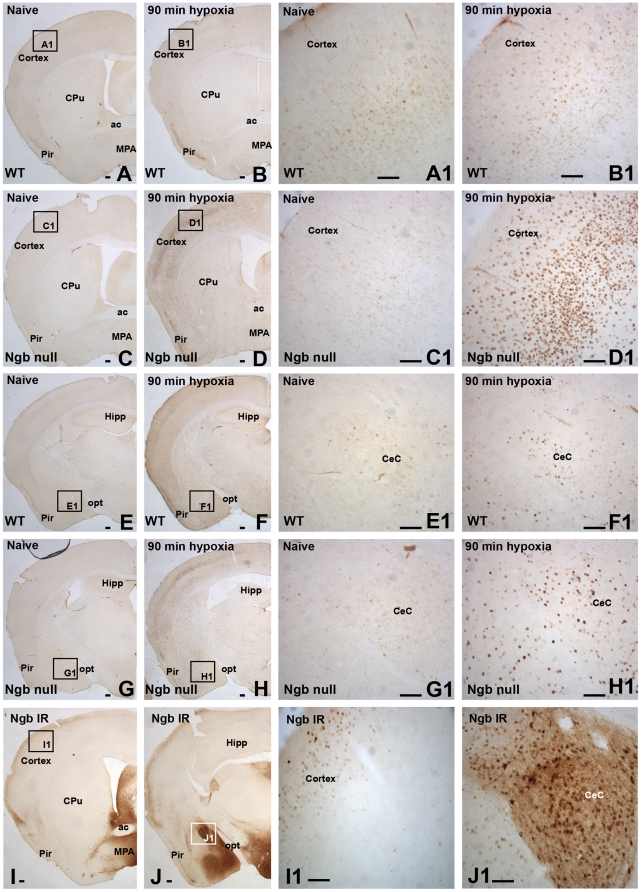
Induction of c-FOS immunoreactivity in response to hypoxia. **A–B**) c-FOS-IR in the anterior forebrain of normoxic (**A**) and hypoxic (**B**) wt mice. The black square inserts of the cerebral cortex are shown in higher magnifications in **A1** and **B1**. A modest induction of c-FOS-IR is apparent in hypoxic wt mice. **C–D**) c-FOS-IR in the anterior forebrain of naive (**C**) and hypoxic (**D**) Ngb-null mice. A substantial hypoxia-dependent increase of c-FOS-IR was observed in Ngb-null mice (**D** and **D1**) when compared to both naive Ngb-null (**C** and **C1**) and hypoxic wt (**B** and **B1**) mice. Similar genotype-dependent differences in c-FOS induction were observed in the posterior forebrain of wt (**E**, **F**) and Ngb-null mice (**G**, **H**). The black square inserts of the amygdaloid complex are magnified in **E1**–**H1**. **I–J**) Ngb-IR in the anterior (**I**) and posterior forebrain (**J**) of naive wt mice. The black square inserts of the cerebral cortex and the amygdaloid complex are shown in higher magnifications in **I1** and **J1**, respectively. Abbreviations: anterior commissure (ac); central amygdaloid nucleus, capsular part (CeC); caudate putamen (CPu); hippocampus (Hipp); medial preoptic area (MPA); optic tract (opt); piriform cortex (Pir). Scale bar 50 µm.

**Figure 4:**
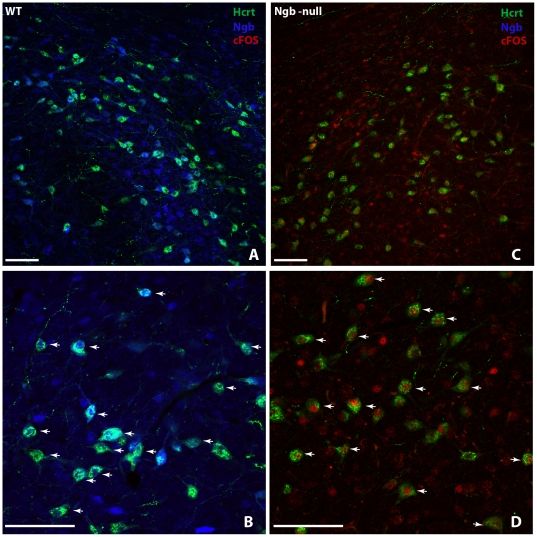
Colocalization of Ngb, Orexin-A and c-FOS immunoreactivity after hypoxia. Immunofluorescence staining of lateral hypothalamus from wt (**A–B**) and Ngb-null (**C–D**) mice after 90 minutes of hypoxia (Orexin-A (Hcrt) – green, c-FOS – red, Ngb – blue). High degree of co-expression of Orexin-A and Ngb-IR (white arrows in B) is observed in wt mice whereas c-FOS is hardly detectable. In Ngb-null mice, most of the Orexin-A-positive neurons are found to co-express c-FOS-IR (white arrows in D). Note the absence of Ngb-IR in the Ngb-null mouse. Scale bar 50 µm.

Hypoxia is known to induce c-FOS expression in many brain areas including those involved in cardio-respiratory regulation [25], [26]. As Ngb-deficiency could be expected to increase neuronal susceptibility to hypoxia, an altered hypoxia-dependent neuronal response would be anticipated in brain regions with high level of endogenous Ngb expression. Therefore we compared the distribution of c-FOS-IR in normoxic and hypoxic mice (Table S1). The level of c-FOS protein was low and comparable in normoxic mice of both genotypes (Figure 3A, C, E, G). Acute hypoxia (90 minutes) induced c-FOS protein expression in both genotypes and within the same brain regions. However, the induction was markedly increased in Ngb-null mice both in terms of intensity and the number of immunoreactive cells (Figure 3D, H). This effect was evident also in brain regions, which normally do not express Ngb. High expression of c-FOS was observed in numerous brain regions including the cerebral cortex (Figure 3D1) and the amygdaloid complex (Figure 3H1). Most of the Orexin-A positive neurons in acute hypoxic Ngb-null mice were found to co-express c-FOS (Figure 4C–D). c-FOS immunofluorescence was hardly detectable in the lateral hypothalamus of hypoxic wild types (Figure 4A–B). No obvious gender-dependent differences in the expression of c-FOS were observed after 90 minutes of hypoxia, except of the tendency for higher c-FOS-IR in the supraoptic nucleus of female mice (Table S1). Immunohistochemical staining of cleaved caspase-3, a marker of activated apoptotic effector mechanisms, was negative after acute and 48 hours hypoxia in both genotypes.

### Transcriptional response to hypoxia

**Figure 5:**
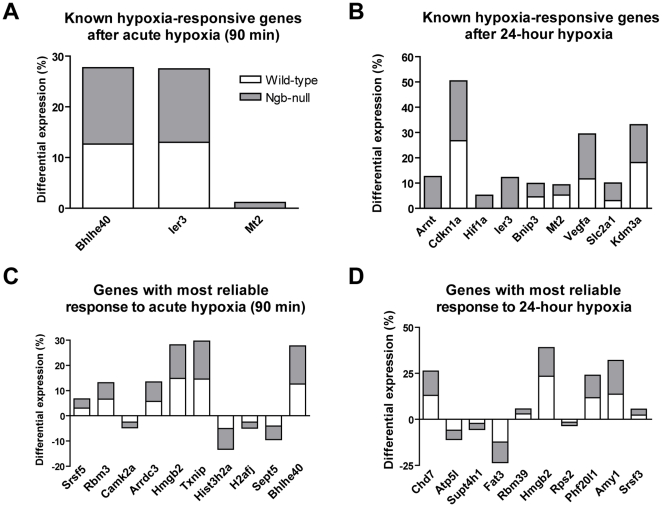
Differential gene expression in response to hypoxia. **A–B**: Differential expression (p<0.05) of several well-known hypoxia-responsive genes after acute (**A**) and 24 hours (**B**) hypoxia according to Affymetrix Mouse Gene 1.0 ST array. Bars represent differential expression between hypoxic and normoxic mice of the respective genotype based on the difference in the mean relative rank of the differentially expressed probes (wt mice - white bars, Ngb-null mice - gray bars). **C–D**: Genes with most reliable differential expression in response to acute (**C**) and 24-hour (**D**) hypoxia based on the product of differential expression p-values in both genotypes. Genes are ordered from left to right by the ascending product of differential expression p-values (i.e. decreasing reliability of differential expression). Bars represent differential expression between hypoxic and normoxic mice of the respective genotype based on the difference in the mean relative rank of the differentially expressed probes (wt mice - white bars, Ngb-null mice - gray bars).

Transcriptional response to hypoxia was evaluated in both genotypes in relation to respective naive group. Gene expression profiling in wt mice identified 517 up-regulated and 347 down-regulated transcripts at 90 minutes and 997 up-regulated and 1862 down-regulated transcripts at 24 hours after the onset of hypoxia (Table S2). In Ngb-null mice there were 404 up-regulated and 364 down-regulated transcripts at 90 minutes and 1535 up-regulated and 1573 down-regulated transcripts at 24 hours of hypoxia (Table S3). The following well-established hypoxia responsive genes were significantly up-regulated (p<0.05) both in the wt and knockout mice: Ier3 (90 min), Bhlhe40 (90 min), Cdkn1A (24 h), Bnip3 (24 h), Mt2 (24 h), Vegfa (24 h), Slc2a1 (24 h), Kdm3a (24 h) (Figure 5A–B). Significant up-regulation of Hif1A during hypoxia was detected only in Ngb-null mice (90 min p = 0.08; 24 h p<0.001). Gnb2l1 also known as Rack1, a hypoxia-regulated receptor of activated protein kinase C 1, which induces HIF1A degradation by competing with HSP90 [27], provided a striking example of down regulation after prolonged hypoxia in Ngb-null mice with 7 out of 9 probes (p<0.001) being significantly down regulated after 24 h of hypoxia whereas none of the probes were differentially expressed after acute hypoxia. Gnb2l1 was significantly down regulated also in wt mice after 24 hours hypoxia (5 out of 9 probes, p<0.01). The hypoxia-dependent up-regulation of the following transcripts was confirmed by quantitative real-time PCR (Figure S1): Ier3 (90 min and 24 h, both genotypes), Cdkn1A (24 h, both genotypes), Hif1A (90 min, both genotypes), Mt2 (24 h, both genotypes), Cygb (90 min and 24 h, Ngb-null mice), Chd7 (90 min and 24 h, Ngb-null mice). In addition, the real-time PCR indicated a higher expression of Hif1A in the Ngb-null mice in relation to wt after 24 hours hypoxia. Extensive overlap between the results from the real-time PCR study and the microarray demonstrate the accuracy of the differential expression estimates obtained using the novel data analysis method employed in this study.

Functional annotation of hypoxia-responsive genes in relation to Kegg pathways [28] produced similar results in both genotypes and highlighted a number of relevant molecular pathways such as “apoptosis” [29], “oxidative phosphorylation” [30], “mTOR signaling“ [31], “VEGF signaling pathway” etc. Cross-validation of hypoxia-responsive genes in our dataset with results from earlier studies (Table S4) established significant enrichment of confirmed (HIF-1)-target genes (extracted from the supplementary materials of [32], hypoxia and ischemia regulated genes (table 4 in [33]), genes regulated in ischemia after hypoxic preconditioning (table 5 in [33]), and transcription and apoptosis related genes that are differentially expressed in the brain after neonatal hypoxia-ischemia (tables 1 and 3 in [29]). Additionally, we observed a significant enrichment of top 30 novel predicted HIF-1 targets obtained from [32] among hypoxia-responsive genes in our experiment.

**Figure 6:**
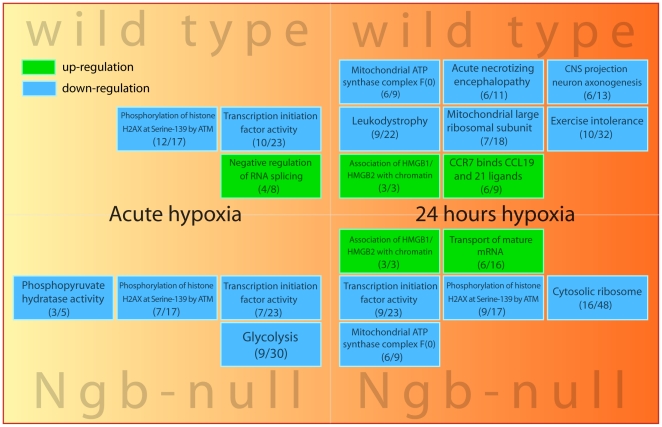
Functional annotation of differentially expressed genes in response to hypoxia. Hypoxia-dependent regulation of gene expression in wt and Ngb-null mice in relation to known molecular pathways. Lists of genes that were differentially expressed between normoxic and acute hypoxic (90 minutes) or 24 hours hypoxic mice were subjected to functional annotation with g:Profiler [23]. Green background represents up-regulation and blue background represents down-regulation of the enriched pathway (p<0.001). The number of differentially expressed genes and the total number of genes in the pathway are indicated below the pathway's name. Due to space limitations, only extensively regulated (at least 30% of the pathway members are differentially expressed) and non-redundant (the pathways are represented by different sets of genes) pathways are presented.

In a more detailed analysis of temporal gene expression dynamics during hypoxia we looked for pathways with significant (p<0.001) enrichment of differentially expressed genes between normoxic and hypoxic wt mice (Table S5) and Ngb-deficient mice (Table S6). Due to space limitations, only extensively regulated (at least 30% of the pathway members are differentially expressed) and non-redundant (the pathways are represented by different sets of genes) pathways are presented in Figure 6. Significant overrepresentation of downregulated genes related to transcription initiation factor activity was observed during acute hypoxia in both genotypes and after 24 hours hypoxia in Ngb-null mice. Downregulation of mitochondrial ATP-synthase complex F(0) genes and upregulation of genes releated to the association of HMGB1/HMGB2 with chromatin were seen after 24 hours hypoxia in both genotypes. Surprisingly, an overrepresentation of downregulated genes related to the glycolysis pathway was seen in Ngb-deficient mice after acute hypoxia.

Finally, in order to identify transcripts, which have the most reliable responses to hypoxia based on the present results, we performed the rank-ordering of differentially expressed transcripts based on the product of their p-values in the wt (Table S7) and Ngb-null mice (Table S8) at respective time points (Figure 5C–D). The five highest-ranking genes after 90 minutes of hypoxia were Srsf5 (serine/arginine-rich splicing factor 5), Rbm3 (RNA binding motif protein 3), Camk2a (calcium/calmodulin-dependent protein kinase II alpha Gene), Arrdc3 (arrestin domain containing 3) and Hmgb2 (high mobility group box 2). The top five hypoxia-responsive genes after 24 hours were Chd7 (chromodomain helicase DNA binding protein 7), Atp5l (ATP synthase, H+ transporting, mitochondrial F0 complex, subunit g), Supt4h1 (suppressor of Ty 4 homolog 1), Fat3 (FAT tumor suppressor homolog 3) and Rbm39 (RNA binding motif protein 39 Gene).

### Genes with differential expression between wt and Ngb-null mice during hypoxia

Only 22 transcripts were identified as differentially expressed between naive wt and naive Ngb-null mice (Table S9). All exhibited lower expression level in the Ngb-null mice. In order to evaluate hypoxia-specific genotype-dependent differences in transcriptional regulation we identified transcripts, which were not differentially expressed between naive mice, but had consistent differential expression between wt and Ngb-null mice both at 90 minutes and at 24 hours after the onset of hypoxia. In Ngb-null mice there was an up-regulation of 181 transcripts and down-regulation of 121 transcripts during hypoxia when compared to respective wt mice (Table S10). The three most significantly (p<0.001) up-regulated transcripts in Ngb-deficient mice during hypoxia were Cspp1 (centrosome and spindle pole associated protein 1), Adi1 (acireductone dioxygenase 1) and Prpf4b (PRP4 pre-mRNA processing factor 4 homolog B). The three most significantly (p<0.001) down-regulated transcripts were Ubc (ubiquitin C), Rplp0 (ribosomal protein, large, P0) and Kidins220 (kinase D-interacting substrate 220).

In order to differentiate transcriptional responses to acute and prolonged hypoxia between genotypes, we identified transcripts, which were differentially expressed between Ngb-null and wt mice only at either 90 minutes or 24 hours of hypoxia. There were 6 transcripts up-regulated in Ngb-null mice in relation to wt at 90 minutes (Table S11). In contrast, there were 344 down-regulated transcripts in Ngb-null mice at 90 minutes. Functional annotation of the transcripts revealed extensive down-regulation of pathways related to signal transduction (e.g. “IkB is ubiquitinated and degraded”, 6 out of 7), metabolism (“glyceraldehyde-3-phosphate dehydrogenase activity”, 8 out of 24) and protein synthesis (“cytosolic small ribosomal subunit”, 13 out of 39) in the Ngb-deficient mice (Table S12). After 24 hours of hypoxia, 205 up-regulated and 13 down-regulated transcripts were detected in Ngb-null mice in relation to corresponding wts (Table S13). Functional annotation analysis of the transcripts did not reveal any extensively differentially expressed pathways (Table S14).

## Discussion

A substantial number of studies have investigated Ngb expression and its potential neuroprotective properties during hypoxia and ischemia (for review please see [34], [35]). Studies in rodents have reported lack of Ngb regulation [7], [11], [36], down-regulation [37] and up-regulation of Ngb [38] following hypoxia. Ischemic rodent models have also yielded conflicting results ranging from no differential expression and no neuroprotective properties in relation to ischemia [6], [39] to up-regulation [10] and significant neuroprotective properties when Ngb expression was modulated by viral gene transfer [13] or up-regulated by transgenic means [12], [15], [40]. In spite of numerous studies on the relation of Ngb and hypoxia/ischemia there remains a lack of consensus as to the role of Ngb protein in neuroprotection. Therefore, in order to increase our understanding of Ngb's potential therapeutic role, we created genetically Ngb-deficient mice and studied their response to hypoxia. We hypothesized that Ngb-deficient mice would be more susceptible to the adverse effects of hypoxia if Ngb protein had substantial neuroprotective properties. The study specifically evaluated the effect of Ngb deficiency on neuronal survival and on transcriptional regulation following acute and prolonged hypoxia.

Ngb-null mice are born in expected rations, they have normal survival to adulthood (age 8 weeks) and they show no obvious differences in overt appearance, body weight and behavior when compared to wt littermates. Hence, Ngb does not seem to be critical for normal development and vitality. To evaluate the effect of Ngb deficiency on neuronal viability after hypoxia, we used Orexin-A-positive and Cygb-positive neurons as surrogate markers for Ngb neurons in the lateral hypothalamus and hindbrain, respectively. We found no statistical difference in the number of these neurons between wt and Ngb-null mice in normoxia or after 24 and 48 hours of hypoxia, suggesting that the loss of Ngb protein does not affect neuronal viability even after severe prolonged hypoxia. However, the apparent lack of cell death does not imply that Ngb-positive and Ngb-deficient neurons are equally resistant to hypoxia. Therefore, we investigated the expression of cleaved caspase-3, a marker of activated apoptotic effector mechanisms, in wt and Ngb-null hypoxic mice. No cleaved caspase-3 immunoreactivity was detected in any of the mice indicating that Ngb deficiency is not sufficient to activate this apoptotic effector molecule during hypoxia. These results are contradictory to reports of decreased survival of Ngb-deficient cells after hypoxia *in vitro* and to the reports on the increase in ischemic infarct size after adenovirus-mediated down-regulation of Ngb *in vivo* [10], [13]. It is important to note however, that the *in vivo* hypoxic conditions differ markedly from both the hypoxia models in the cell culture and from the models of cerebral ischemia. Specifically, the oxygen concentration in the microenvironment surrounding neurons during *in vivo* experimental hypoxia is unknown while intact cerebral blood flow still provides cells with nutrients and serum, which is not the case in ischemia. In addition, one cannot exclude that inborn deficiency in Ngb function might induce compensatory mechanisms, which are able to substitute for the lack of Ngb during normal and hypoxic conditions. In the aforementioned studies the experimental down-regulation of Ngb was acute, which is unlikely to allow for a substituting effect. However, since the mice used in the present study had no prior experience of hypoxia there seems to be little reason to assume that the brains of Ngb-null mice had adapted to low oxygen availability. It is therefore more likely that Ngb is not necessary for neuronal survival even after severe prolonged hypoxia.

Although Ngb deficiency does not seem to affect neuronal survival during hypoxia, we found a widespread and more pronounced increase in the number of c-FOS-IR neurons in Ngb-null mice than in wt mice after acute hypoxia. A hypothesis that Ngb deficiency lowers the threshold for hypoxia-induced gene expression response is also supported by the global analysis of gene expression, which identified the increased expression of Hif1A and its heterodimer partner Arnt in Ngb-null mice after 24 hours of hypoxia.

Our results from the large-scale analysis of gene expression are well in line with previous reports (see [41], for a review) and they confirmed the hypoxia-dependent regulation of pathways related to, for example, apoptosis, cell growth (“mTOR signaling”), synthesis of ATP (“oxidative phosphorylation”) and angiogenesis (“VEGF signaling pathway”) in both genotypes. Based on a broader categorization of the differentially regulated pathways we will focus the discussion on glucose metabolism, chromatin remodeling and RNA processing.

Most cells produce ATP by mitochondrial oxidative phosphorylation under normoxic conditions. However, oxidative phosphorylation is inefficient under hypoxic conditions, and adaptation to hypoxic stress is achieved by the down-regulation of mitochondrial oxygen consumption and by increased reliance on anaerobic glycolysis for energy production [42]. Consequently, the largest functional group of genes regulated by HIF1 in many cell types are associated with glucose metabolism [43]. We identified a coordinated down-regulation of genes encoding subunits of mitochondrial ATP synthase complexes F0 and F1, several cytochrome c oxidase subunits, NADH dehydrogenase subunits and an up-regulation of glucose transporter 1 (Slc2a1) gene in both genotypes after 24-h hypoxia (Table S15). Interestingly, an up-regulation of monocarboxylate transporter 4 gene (Slc16a4) encoding a plasma membrane protein that removes lactate, the end product of anaerobic glycolysis, from the cell, was detected after exposure to hypoxia only in Ngb-null mice. More surprisingly, a down-regulation of the following genes of the glycolysis pathway was detected in Ngb-null mice after acute hypoxia: Eno1 (enolase 1, alpha non-neuron), Eno2 (enolase 2, gamma neuronal), Gapdh (glyceraldehyde-3-phosphate dehydrogenase), Pfkl (phosphofructokinase, liver, B-type Gene) and Aldoa (aldolase A, fructose-bisphosphate). Of those, Gapdh was down-regulated also after 24 hours hypoxia. Recent evidence indicating that hypoxia-dependent post-translational protein modification by SUMO1 (small ubiquitin related modifier 1) may activate the glycolytic pathway [44], prompted us to look at the differential expression of the 3 genes of the SUMO protein family and SUMO/sentrin specific protease genes, which encode proteins which can reverse SUMOylation [45]. We detected an up-regulation of Sumo3 and Sumo2 after acute hypoxia in the wt and Ngb-deficient mice, respectively. Sumo1 and Sumo2 were up-regulated in the wt mice, but not in the Ngb-deficient mice after 24-h hypoxia. Down-regulation of Senp8 (SUMO/sentrin specific protease family member 8) was detected in wt mice after both acute and 24-h hypoxia. These observations point to a more widespread hypoxia-dependent regulation of the SUMOylation pathway genes in the wt mice than in the Ngb-deficient mice. Altogether, the observations cited above suggest that the transcriptional down-regulation of mitochondrial activity during 24 hours hypoxia is intact in Ngb-null mice, but the regulation of the glycolytic pathway and SUMO-related genes is altered.

Hypoxia-dependent regulation of chromatin remodeling pathways was evident from the down-regulation of genes with tags “nucleosome assembly” and “Phosphorylation of histone H2AX at Serine-139 by ATM” in both genotypes after acute hypoxia and in Ngb-null mice also after 24 hours hypoxia. In contrast, there was an up-regulation of genes with tags “chromatin modification” and “Association of HMGB1/HMGB2 with chromatin” after 24 hours hypoxia in both genotypes. Inspection of the implicated genes (Table S16) revealed numerous core histones from histone cluster 1 and 2 genes, ATP-dependent chromatin remodelers (Smarca4, Smarca5, Chd4, Chd7, Chd8), genes of heterochromatin- (Cbx3) and euchromatin-associated proteins (Hmgb1, Hmgb2, Hmgb3, Hmgn3, Pole3), histone methyl transferases (Mll5, Setdb2, Ehmt1), histone demethylases (Jmjd6, Kdm2a, Kdm3a, Kdm4a, Kdm4c, Kdm5a, Kdm6a) and RNA polymerase 2 associated factors (Paf1, Leo1) etc. Although studies on hypoxia-dependent chromatin remodeling are rather scarce [46], hypoxia-dependent modification of histones has been documented in a number of cell lines [47], [48], [49], [50], [51]. Our results demonstrate the down-regulation of core histone genes after acute hypoxia and the up-regulation of genes encoding histone-modifying proteins after 24 hours hypoxia in both genotypes. Incidentally, a study of endothelial nitric oxide gene has documented the eviction of histones from its core promoter and a subsequent reincorporation of histones with altered post-translational modifications in response to hypoxia [49]. It is possible that a similar hypoxia-dependent resetting of histone modification patterns occurs in the brain and that it revolves in stages, which coincide with the down-regulation of core histones, followed by the up-regulation of histone modification pathways. The importance of histones in adapting to a hypoxic environment is also suggested by a comparative exome-wide study of SNP frequencies in ethnic Tibetans where 3 out of the 30 most significant loci were linked to genes in histone cluster 1 [52]. Finally, among the 60 transcripts with most reliable response to hypoxia, we found several which encode proteins regulating chromatin structure (Hist3h2a, H2afj, Chd7 and Hmgb2).

Up-regulation of mRNA processing and mRNA metabolic pathways (tags “Formation and Maturation of mRNA Transcript”, “mRNA metabolic process”, “mRNA processing”, “mRNA Splicing”) was detected in both genotypes after 24 hours hypoxia, but not after acute hypoxia. The main post-transcriptional mechanisms which regulate gene expression during hypoxia appear to be mRNA turnover and translational control [53]. We detected, for example, the up-regulation of Ptbp2 (known to promote Hif1a mRNA translation and stabilize Vegf mRNA during hypoxia, see [53] for overview), genes of several heterogeneous nuclear ribonucleoproteins (regulate translation by affecting mRNA secondary structure), various RNA binding motif proteins (related mostly to mRNA splicing) and THO complex genes (required for normal transcriptional elongation and recruitment of splicing factors) [54]. These observations suggest that post-transcriptional processes might play a prominent role in the adaptation of the brain tissue to prolonged hypoxia.

### Conclusion

The present study demonstrates that the lack of neuroglobin in mice alters the response of c-FOS, Hif1A and the regulation of the glycolytic pathway genes whereas there is no effect on neuronal and organismal survival rate or behavior during severe acute and prolonged hypoxia. Analysis of global gene expression in both genotypes suggests chromatin remodeling and mRNA metabolism to be among the key regulatory mechanisms when adapting to prolonged hypoxia. Further studies are necessary to clarify the role of neuroglobin in the regulation of cellular metabolism.

## Materials and Methods

### Ngb deficient mice

*Ngb* knock-out mouse model was created by genOway (Lyon, France) under the project no. genOway/SST/HSA1-*Ngb*/260307. In brief, homology regions covering 5.9 kb upstream of Ngb exon 2 and 2 kb downstream of exon 3 were subcloned from a miniBAC clone #1641C8 from 129Sv/Pas mouse genomic BAC library. FRT-flanked Neo resistance positive selection cassette was inserted downstream of exon 3 and two loxP sites were introduced upstream of exon 2 and downstream of exon 3, respectively (Figure 1A). The targeting construct was introduced into the mouse genome by homologous recombination in 129Sv/Pas embryonic stem (ES) cells (genOway, Lyon, France) and recombinant clones were isolated by resistance to G418. Germline chimeras were obtained by injection of recombinant ES cells into C57BL/6J blastocysts (genOway, Lyon, France). Chimeric mice were crossed with C57Bl/6J mice to obtain F1 mice carrying the recombined allele containing the floxed Ngb allele and Neo selection cassette. These mice were mated with Flp recombinase-expressing C57Bl/6J Flp mice (genOway, Lyon, France) to remove the Neo resistance cassette and generate a line of Neo-excised floxed mice. Heterozygous Neo-excised floxed mice were then crossed with C57Bl/6J mice expressing Cre recombinase under the cytomegalovirus promoter (genOway, Lyon, France), which resulted in the genomic deletion of *Ngb* exons 2 and 3 from the floxed allele during the early stages of embryonic development. The heterozygous Ngb-deficient founder mice (F3 generation of C57Bl/6J backcross) were further backcrossed with wild-type C57Bl/6J mice for 6 generations and the offspring were monitored for the absence of *Cre*-allele by PCR from tail biopsies (please see the next section, for primer sequences). Thus, the Ngb-deficient mouse strain used for this study was negative for the Cre allele and it was backcrossed to the C57Bl/6J genomic background for 9 generations.

### Genotyping

Tail-clip genotyping was performed using the following primer sets in 0.2 µM concentrations: 5′-CTCTCAGGCGTGAGGTAAGG-3′, 5′-CTGGCTCCCACTGTATTGGT-3′, 5′-CCCAGA TTCTGATACCCAC-3′ and 5′-GCACTCCTTGCTGAGACCTAG-3′ using 1× DyNazyme reaction buffer and DyNazyme I polymerase (cat. No. F-500S Finnzymes, Finland), 0.25 µM dNTP (Amresco, USA). The PCR reaction was run as follows: 94°C 2 min, 92°C 1 min, 35 cycles of 60°C 1 min, 72°C 30 sec, 92°C 1 min, 60°C 1 min, 72°C 10 min. The PCR products were run on a 1% agarose gel where bands of approximately 2000 bp and 600 bp indicate wt (wt), 600 bp and 180 bp bands indicate heterozygous, and 180 bp band indicates homozygous Ngb-deficient genotype (Ngb-null). For detection of the *Cre*-allele the following primers were used 5′-AAA CGT TGA TGC CGG TGA ACG-3′ and 5′-CAG CCA CCA GCT TGC ATG AT-3′ giving an approximately 600 bp product in the presence of the C*re*-allele.

### Southern blotting

Southern blotting was performed by genOway according to their standard protocol. For confirmation of 3′ recombination of the floxed Ngb allele genomic DNA from heterozygous Neo-excised floxed mice was digested by BamHI-SpeI and tested with the external 3′ probe, generated by PCR on genomic DNA using the following primer pair (5′-3′): 11597PRO-HSA1 GTCCCCCCGCCCTGTCCTCTTTGTCTGC and 11598PRO-HSA1 CCCATGGGGACAGACCATAG (product size 532 bp). This screening, based on the BamHI-SpeI digestion of the genomic DNA should lead to the detection of the following specific 3′ DNA fragments: wild-type 11153 bp, recombined 7765 bp, floxed (Flp-mediated Neo-excised) 9457 bp.

### Antibodies

Primary and secondary antibodies used for Western blotting and immunohistochemistry (IHC) are listed in Table S17 and Table S18, respectively.

### Western blotting

One male mouse from each genotype was euthanized by decapitation. The brain was rapidly removed and, on ice, cut sagittally dividing it in two hemispheres. The tissue was frozen on dry ice and stored at −80°C until the protein extraction. Following the addition of 1 mL ice-cold Cell Extraction Buffer (Invitrogen Carlsbad, CA, USA) supplemented with 1% Halt Phosphatase Inhibitor Cocktail (Pierce, Rockford, IL, USA) and protease inhibitors (Roche Mini EDTA-free Complete® tablet) to the frozen brain tissue, it was homogenized with the aid of 10 strokes of a pellet pestle and a sterile scalpel. After 30 minutes of incubation on ice, the extracts were cleared of insoluble material at 15000× *g* for 10 min at 4°C and the supernatant was transferred to clean tubes and stored at −80°C.

All reagents and equipment used for electrophoresis and transfer of proteins were used according to manufacturers' instructions regarding the NuPAGE® system (Invitrogen). Frozen brain lysate was briefly thawed on ice, followed by the addition of equal amount of 2× SDS sample buffer (100 mM Tris (pH 6,8), 8% SDS, 24% glycerol, 80 mM HCl and 0,025% Coomasie brilliant blue) freshly supplemented with 1× NuPAGE Reducing agent (Invitrogen, Carlsbad, CA, USA) and incubated for 10 min at 70°C. Equal amounts (20 µL) of each sample (wt, heterozygote and Ngb-null) were loaded on a 10% Bis-Tris gel (Invitrogen, Carlsbad, CA, USA) and immunoblotted for Ngb as described below. Recombinant Ngb [18] was used as a positive control. Immunoblotting was conducted over night at 4°C with Ngb antiserum or actin as loading control. Immunoreactivity was detected with donkey anti-guinea pig IgG and goat anti-mouse IgG horseradish peroxidase-conjugated secondary antibodies. Protein bands were visualized with enhanced chemiluminescence according to manufacturer's protocol (Western Lightning Plus-ECL, PerkinElmer, Waltham, MA, USA). Photographs of developed films were adjusted for contrast and brightness in Adobe Photoshop CS2.

### Animals

All animal experiments were performed in accordance to the rules of Dyreforsoegstilsynet, Ministry of Justice, Denmark, who issued the license number 2010/561-1834 to AHS thereby approving the study. At the time of the experiment the age of the mice was 16–18 weeks. 31 male and 46 female mice were exposed to hypoxia in the experiments. The following groups of wt and Ngb-null mice were exposed to hypoxia: 90 min (10 male/8 female wt and 5 male/5 female Ngb-null), 180 min (5 male wt and 3 male Ngb-null), 24 hours (5 female wt and 4 female Ngb-null), 48 hours (5 female wt and 5 female Ngb-null), 72 hours (4 male wt and 4 male Ngb-null) and 96 hours (7 female wt and 7 female Ngb-null). Since no major sex-dependent differences in global gene expression response to prolonged hypoxia have been reported in the adult rodent's brain only female mice were used for the microarray gene expression study and quantitative real-time PCR analysis: 3 normoxic wt, 5 normoxic Ngb-null, 5 90-minutes hypoxic wt, 5 90-minutes hypoxic Ngb-null, 5 24-hours hypoxic wt and 4 24-hours hypoxic Ngb-null. Each brain was divided into two halves, one half for gene expression array and quantitative real-time PCR analysis and the other half for IHC. For c-FOS IHC, the following mice were used: 4 normoxic wt mice (2 males, 2 females), 3 normoxic Ngb-null mice (2 males, 1 female), 9 90-minutes hypoxic wt mice (5 females, 4 males), 9 90-minutes hypoxic Ngb-null mice (5 females, 4 males); 5 180-minutes hypoxic wt (all males) and 3 180-minutes hypoxic Ngb-null (all males).

### Hypoxia model

The hypoxia chambers used for the present study were 57×32×35 cm (LxDxW) rigid plastic cages with a glass cover. Mice were housed in the hypoxia chambers for two days prior to the experiment with free access to food and water. Hypoxia was initiated by flushing the chamber with a premixed gas mixture of 93% N_2_ and 7% O_2_ (AGA, Denmark). Behavior was monitored several times daily. With the exception of one wt mouse, all mice survived the hypoxia exposure.

### Immunohistochemistry

Mice used for IHC and gene expression analysis were euthanized by gentle cervical dislocation. The brains were rapidly removed and either immersion fixed at 4°C for three days in phosphate buffered 10% formalin (FA) or snap frozen on dry ice and stored at −80°C until use. For IHC the brains were stored in FA at 4°C for three days, cryoprotected in 30% sucrose-PBS for 5 days, frozen and sectioned in 40 µm thick coronal slices in four series. The IHC was performed according to the protocols previously described [9], [19]. For single IHC staining Ngb was detected by using a polyclonal guinea pig anti-Ngb antibody. c-FOS and cleaved-caspase-3 were detected with polyclonal antibodies raised in rabbit. The guinea pig Ngb antibody was visualized by a (Fab)_2_ fragment of donkey anti-guinea pig antibody conjugated to biotin and the rabbit c-FOS and cleaved-caspase-3 were detected by biotinylated donkey anti-rabbit antibody (Fab)_2_ fragment in combination with Avidin-Biotin-peroxidase Complex (ABC) (Vector labs, Burlingame, CA, USA), followed by 0.05% diaminobenzidine (DAB). For double and triple immunostaining Ngb was detected using the guinea pig polyclonal antibody in combination with polyclonal rabbit anti-Cygb, goat anti-Orexin-A or rabbit anti-c-FOS and visualized by appropriate secondary antibodies conjugated to fluorophore. As negative controls the primary antibodies were omitted, which eliminated all staining from the respective secondary antibodies. No Ngb-IR was seen in the brain of Ngb-null mice.

### Effect of Ngb deficiency on the number of orexin and Cygb-IR neurons after hypoxia

The effect of Ngb deficiency on neuronal survival was estimated by immunohistochemical staining of markers of Ngb neurons during normoxia and after hypoxia (24 h and 48 h) in the lateral hypothalamus (LH, Orexin-A as surrogate marker) and in two hindbrain nuclei, the pedunculopontine tegmental nucleus and the laterodorsal tegmental nucleus (PPTg and LDTg, Cygb as surrogate marker). The number of Orexin-A-immunoreactive (IR) and Cygb-IR neurons and double stained neurons with Ngb-IR was counted in wt (n = 3–6) and Ngb-null (n = 4–6) mice at three levels of LH and two levels of PPTg and LDTg (distance between sections was 160 µm) from digital photomicrographs using ImageJ (http://rsbweb.nih.gov/ij/) cell counter plugin as described in [20]. Statistical comparisons were made using Mann Whitney test.

### Microarray analysis

Microarray analysis was performed at Rigshospitalet Microarray Center, Copenhagen, Denmark (www.rhmicroarray.com). In short, 50 ng of total RNA from each sample was amplified using the WT-Ovation Pico RNA Amplification System (Nugen, San Carlos, CA, USA) according to manufactures instructions. Double-stranded cDNA was generated using the WT-Ovation Exon module followed by biotin labeling with the FL-Ovation cDNA Biotin Module V2. The labeled samples were hybridized to the Mouse Gene 1.0 ST GeneChip array (Affymetrix, Santa Clara, CA, USA). The arrays were washed and stained with phycoerytrin conjugated streptavidin (SAPE) using the Affymetrix Fluidics Station® 450, and the arrays were scanned in the Affymetrix GeneArray® 3000 scanner to generate fluorescent images, as described in the Affymetrix GeneChip® protocol. Cell intensity files (CEL files) were generated in the GeneChip® Command Console® Software (AGCC) (Affymetrix, Santa Clara, CA, USA).

### Microarray Data Analysis

The microarray data produced in this study is MIAME compliant (http://www.mged.org/Workgroups/MIAME/miame.html) and it has been submitted to the ArrayExpress database (www.ebi.ac.uk/arrayexpress/) under accession No. E-MTAB-726. Probe-level expression data was obtained from CEL files and analyzed using R (www.r-project.org), according to a novel non-parametric differential expression analysis method. The method is based on two basic assumptions about the oligonucleotide gene expression array. The first assumption is the degree to which probe-level signals reflect the expression of a given target sequence is dependent on the sequence similarity between the probe and the target. Second, it is assumed that probes reporting the expression level of the same target accurately possess similar differential expression profiles in the experiment. The method makes use of the hypergeometric probability function to estimate the likelihood that a given differential expression profile can be attributed to the transcript in question after counting the number of probes with statistically significant differential expression profiles and identical target transcripts. The workflow implements the following steps:

1. alignment of all probe sequences represented on the Affymetrix Mouse Gene 1.0 ST array to the mouse transcriptome (probes which do not produce any alignment hits are discarded)
2. probe-level normalization of expression signals by relative ranking on each array to ensure that probe signal distributions are identical on all arrays
3. probe-level differential expression analysis using Mann Whitney test
4. clustering of probes based on their differential expression profiles in the experiment
5. estimation of statistical significance for the over-representation of probes which target the same transcript among those with identical differential expression profile using the following formula:
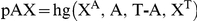

pAX the likelihood of being mistaken when attributing expression profile A to transcript X

hg the hypergeometric probability function

X^A^ the number of probes with expression profile A that have alignment hit(s) to transcript X

A the number of probes with expression profile A

T the total number of probes in the analysis

X^T^ the number of probes that have alignment hit(s) to transcript X

The differential expression profile of each probe was based on the comparisons between Ngb-null and wt groups at corresponding time points during hypoxia (0, 90 min, 24 h), and on comparisons between untreated wts with hypoxic wts (90 min and 24 h), and between untreated Ngb-null mice and hypoxic Ngb-null mice (90 min and 24 h). Based on each comparison the probe was assigned either letter ‘H’ to denote higher expression in the experimental group in relation to control (p< = 0.05, Mann Whitney test upper-tail probability), letter ‘L’ to denote lower expression in the experimental group in relation to control (p< = 0.05, Mann Whitney test lower-tail probability), or ‘E’ to denote the absence of differential expression (p>0.05). By combining the letters from related comparisons each probe was assigned an expression string describing its differential expression dynamics in the experiment. After clustering probes according to the expression string identity the formula given above was used to estimate the pA^X^ for all transcripts X in relation to all expression profiles A. After adjusting the p-values to account for multiple hypotheses testing [21], transcripts with adjusted p-values less than 0.05 were considered as differentially expressed.

### Alignment of probe sequences to the mouse transcriptome

Sequences of 1,102,500 probes represented on the Affymetrix Mouse Gene 1.0 ST array were obtained from the Affymetrix homepage (www.affymetrix.com) and aligned to the Ensembl build of the mouse transcriptome (NCBIM37.56) using BLAT application [22]. At least a 22-nucleotide ungapped alignment between the probe and the transcript's minus strand was required to produce an alignment hit. The number of probes with alignment hits was 305,499 (28%).

### Functional annotation analysis and comparison of gene lists

Detailed functional annotation analysis was performed with g:Profiler [23], which is available at http://biit.cs.ut.ee/gprofiler/ (all analysis parameters were set to default values). Comparison of differentially regulated genes during hypoxia in relation to Kegg pathways (http://www.genome.jp/kegg/pathway.html) and in relation to gene lists from previously published reports was performed by summing the hypergeometric probability densities obtained by dhyper function in R as follows:
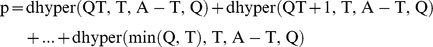
where QT is the size of the intersection between the differentially regulated genes and the target gene list, T is the number of genes in the target list, A is the total number of mouse genes, Q is the number of differentially regulated genes and min(Q,T) is T if T<Q or otherwise it is Q.

### Quantitative Real-Time PCR

500–1000 ng total RNA was reverse transcribed using superscript III Reverse Transcriptase kit according to the manufactures instructions (Invitrogen, Carlsbad, CA). The mix was subsequently diluted 10 fold. The expression of the transcripts was quantitated using the following Taqman (Applied Biosystems) assays: actin beta (Actb) (Mm01205647_g1), chromodomain helicase DNA binding protein 7 (Chd7) (Mm01219540_m1), cyclin-dependent kinase inhibitor 1A (P21) (Cdkn1a) (Mm00432448_m1), cytoglobin (Cygb) (Mm00446071_m1), immediate early response 3 (Ier3) (Mm00519290_g1), metallothionein 2 (Mt2) (Mm00809556_s1) and Neuroglobin (Ngb) (Mm00452101_m1). The expression was normalized to the expression of beta-actin using the delta-delta-CT method [24].

## Supporting Information

Figure S1 **Confirmation of differential gene expression by quantitative real-time PCR (RT-QPCR).** Data is plotted as mean +/− standard error. Unless indicated otherwise, an asterisk denotes a statistically significant difference in gene expression between the group bearing the asterisk and the naive group of the same genotype. * p<0.05, ** p<0.001 (Mann-Whitney test). Genotypes: wild-type (white bars), Ngb-null (black bars).
(EPS)

Table S1 **Quantification of c-FOS immunoreactivity in naive, 90 minutes hypoxic and 24 hours hypoxic wt mice and Ngb-deficient mice.**
(XLS)

Table S2 **Differential expression of transcripts after 90 minutes and 24 hours of hypoxia in wt mice.** Differential expression is in relation to naive mice of the same genotype. Data is presented on 4 worksheets entitled according to their contents. Where appropriate, description of the column has been provided as a comment in the column heading.
(XLS)

Table S3 **Differential expression of transcripts after 90 minutes and 24 hours of hypoxia in Ngb-deficient mice.** Differential expression is in relation to naive mice of the same genotype. Data is presented on 4 worksheets entitled according to their contents. Where appropriate, description of the column has been provided as a comment in the column heading. Due to space limitations, only transcripts with adjusted p-value<0.5 are listed.
(XLS)

Table S4 **Enrichment of differentially expressed genes in relation to KEGG pathways and previously published data.** Several of the known hypoxia-responsive pathways are indicated in red. Gene lists extracted from publications referenced below the table are indicated in bold.
(XLS)

Table S5 **Functional annotation of differentially expressed genes in wt mice by g:Profiler.** Data is presented on 4 worksheets entitled according to their contents. Clicking on a hyperlink in the Q&T column will create a new g:Profiler query using the genes that triggered the annotation.
(XLS)

Table S6 **Functional annotation of differentially expressed genes in Ngb-deficient mice by g:Profiler.** Data is presented on 4 worksheets entitled according to their contents. Clicking on a hyperlink in the Q&T column will create a new g:Profiler query using the genes that triggered the annotation.
(XLS)

Table S7 **Transcripts with most reliable response to hypoxia in wt mice.** The table was created by rank-ordering differentially expressed transcripts based on the ascending product of their adjusted p-values after 90 minutes and 24 hours of hypoxia.
(XLS)

Table S8 **Transcripts with most reliable response to hypoxia in Ngb-deficient mice.** The table was created by rank-ordering differentially expressed transcripts based on the ascending product of their adjusted p-values after 90 minutes and 24 hours of hypoxia.
(XLS)

Table S9 **Differentially expressed transcripts between naive wt and naive Ngb-null mice.** Where appropriate, description of the column has been provided as a comment in the column heading.
(XLS)

Table S10 **Transcripts with consistent differential expression between wt and Ngb-null mice at 90 minutes and at 24 hours after the onset of hypoxia, but not during normoxia.**
(XLS)

Table S11 **Transcripts differentially expressed between wt and Ngb-deficient mice only after 90 minutes of hypoxia.** Where appropriate, description of the column has been provided as a comment in the column heading.
(XLS)

Table S12 **Functional annotation of transcripts from Table S11 using g:Profiler.** Clicking on a hyperlink in the Q&T column will create a new g:Profiler query using the genes that triggered the annotation.
(XLS)

Table S13 **Transcripts differentially expressed between wt and Ngb-deficient mice only after 24 hours of hypoxia.** Where appropriate, description of the column has been provided as a comment in the column heading.
(XLS)

Table S14 **Functional annotation of transcripts from Table S13 using g:Profiler.** Clicking on a hyperlink in the Q&T column will create a new g:Profiler query using the genes that triggered the annotation.
(XLS)

Table S15 **Differentially expressed genes in response to hypoxia that are related to ATP synthesis.**
(XLS)

Table S16 **Differentially expressed genes in response to hypoxia that are related to chromatin remodeling.**
(XLS)

Table S17 **Primary antibodies used in this study.**
(DOC)

Table S18 **Secondary antibodies used in this study.**
(DOC)
