# Supplementary material for: Neuroglobin-Deficiency Exacerbates Hif1A and c-FOS Response, but Does Not Affect Neuronal Survival during Severe Hypoxia In Vivo
Source: PLoS One. 2011 Dec 2;6(12):e28160. doi: 10.1371/journal.pone.0028160 (PMC3229544; doi:10.1371/journal.pone.0028160)
Supplement: Table S17 — Primary antibodies used in this study. (DOC) [file pone.0028160.s018.doc]

| Antigen | Antibody | Working dilution | Source |
| --- | --- | --- | --- |
| Actin | Mouse monoclonal. Immunogen: an SDS extract from human myocardium | 1:2000 | Calbiochem, Merck Biosciences, Nottingham, UK. Code CP64 |
| Caspase-3 | Rabbit, polyclonal. Immunogen: a synthetic peptide corresponding to amino-terminal residues adjacent to (Asp175) in human caspase-3. | 1:1000 | Cell Signaling Technolgy, Beverly, MA, USA. Code 9661 |
| cFOS | Rabbit, polyclonal. Immunogen: a synthetic peptide (SGFNADYEASSSRC) corresponding to amino acids 4-17 of human c-Fos | 1:1000-3000 | Calbiochem, Merck Biosciences, Nottingham, UK. Code PC38 |
| Cytoglobin | Rabbit, polyclonal. Immunogen: purified recombinant human Cytoglobin. | 1:30.000 | In house. Code 5092/6. The immunogen was a gift from Dr. Sylvia Dewilde, University of Antwerpen |
| Neuroglobin | Rabbit, polyclonal  Immunogen: purified recombinant mouse Neuroglobin | 1:1000-5000 | In house Code G. The immunogen was a gift from Dr. Sylvia Dewilde, University of Antwerpen |
| Orexin-A | Goat, polyclonal  Immunogen: C-terminus of orexin-A of human origin. | 1:1000 | Santa Cruz Biotechnology, Inc. CA, USA. Code sc-8070 |
