# Supplementary material for: Neuroglobin-Deficiency Exacerbates Hif1A and c-FOS Response, but Does Not Affect Neuronal Survival during Severe Hypoxia In Vivo
Source: PLoS One. 2011 Dec 2;6(12):e28160. doi: 10.1371/journal.pone.0028160 (PMC3229544; doi:10.1371/journal.pone.0028160)
Supplement: Table S18 — Secondary antibodies used in this study. (DOC) [file pone.0028160.s019.doc]

| Species | Reactivity | Working dilution | Conjugate | Source |
| --- | --- | --- | --- | --- |
| Donkey | Rabbit | 1:2000 | Biotin | Jackson Immunoresearch Laboratories, Baltimore, PA, USA. Code 706-066-152 |
| Donkey | Guinea pig | 1:2000 | Biotin | Jackson Immunoresearch Laboratories, Baltimore, PA, USA. Code 706-066-148 |
| Donkey | Rabbit | 1:500 | DyLight-488 | Jackson Immunoresearch Laboratories, Baltimore, PA, USA. Code 706-486-152 |
| Donkey | Rabbit | 1:500 | DyLight-594 | Jackson Immunoresearch Laboratories, Baltimore, PA, USA. Code 706-506-148 |
| Donkey | Goat | 1:500 | DyLight-488 | Jackson Immunoresearch Laboratories, Baltimore, PA, USA. Code 705-486-147 |
| Donkey | Guinea pig | 1:100 | DyLight-649 | Jackson Immunoresearch Laboratories, Baltimore, PA, USA. Code 706-496-148 |
| Donkey | Guinea pig | 1:2500 | HRP | Jackson Immunoresearch Laboratories, Baltimore, PA, USA. Code 706-036-148 |
| Goat | Mouse | 1:2500 | HRP | Dako, Glostrup, Denmark. Code P0447 |
